# Mitochondrial Protein-Coding Gene Expression in the Lizard *Sphenomorphus incognitus* (Squamata:Scincidae) Responding to Different Temperature Stresses

**DOI:** 10.3390/ani14111671

**Published:** 2024-06-03

**Authors:** Lemei Zhan, Jingyi He, Siqi Meng, Zhiqiang Guo, Yuxin Chen, Kenneth B. Storey, Jiayong Zhang, Danna Yu

**Affiliations:** 1 College of Life Sciences, Zhejiang Normal University, Jinhua 321004, China; 2022205000889@zjnu.edu.cn (L.Z.);; 2 Department of Biology, Carleton University, Ottawa, ON K1S5B6, Canada; ken.storey@gmail.com; 3 Key Lab of Wildlife Biotechnology, Conservation and Utilization of Zhejiang Province, Zhejiang Normal University, Jinhua 321004, China

**Keywords:** mitochondrial genome expression, lizard, low-temperature stress, high-temperature stress

## Abstract

**Simple Summary:**

In order to withstand extreme temperatures, organisms can change their behavior, physiology, and biochemistry prior to or in reaction to environmental variations. This allows them to enhance their chances of survival under challenging conditions. However, lizards are ectothermic, relying on environmental temperature for body heat and making them highly susceptible to climate change. Mitochondria play a crucial role in maintaining body temperature, regulating metabolic rates, and preventing cellular oxidative damage. If mitochondrial respiratory chain complexes become damaged or disrupted, this can be fatal. Mitochondrial DNA also serves as a sentinel for cell stress. Here, we focused on the lizard *Sphenomorphus incognitus* (Squamata:Scincidae) to explore the expression of the 13 mitochondrial protein-coding genes (PCGs) responding to a wide range of temperatures: 4 °C, 8 °C, 25 °C (control group), 34 °C, and 38 °C for 24 h. The aim was to explore the potential cold resistance and heat tolerance mechanisms of lizards by examining the expression of mitochondrial genes under gradient temperature conditions, as a means to assess the adaptability of lizards in the current context of climate change.

**Abstract:**

In the context of global warming, the frequency of severe weather occurrences, such as unexpected cold spells and heat waves, will grow, as well as the intensity of these natural disasters. Lizards, as a large group of reptiles, are ectothermic. Their body temperatures are predominantly regulated by their environment and temperature variations directly impact their behavior and physiological activities. Frequent cold periods and heat waves can affect their biochemistry and physiology, and often their ability to maintain their body temperature. Mitochondria, as the center of energy metabolism, are crucial for maintaining body temperature, regulating metabolic rate, and preventing cellular oxidative damage. Here, we used *RT*-qPCR technology to investigate the expression patterns and their differences for the 13 mitochondrial PCGs in *Sphenomorphus incognitus* (Squamata:Scincidae), also known as the brown forest skink, under extreme temperature stress at 4 °C, 8 °C, 34 °C, and 38 °C for 24 h, compared to the control group at 25 °C. In southern China, for lizards, 4 °C is close to lethal, and 8 °C induces hibernation, while 34/38 °C is considered hot and environmentally realistic. Results showed that at a low temperature of 4 °C for 24 h, transcript levels of *ATP8*, *ND1*, *ND4*, *COI*, and *ND4L* significantly decreased, to values of 0.52 ± 0.08, 0.65 ± 0.04, 0.68 ± 0.10, 0.28 ± 0.02, and 0.35 ± 0.02, respectively, compared with controls. By contrast, transcript levels of *COIII* exhibited a significant increase, with a mean value of 1.86 ± 0.21. However, exposure to 8 °C for 24 h did not lead to an increase in transcript levels. Indeed, transcript levels of *ATP6*, *ATP8*, *ND1*, *ND3*, and *ND4* were significantly downregulated, to 0.48 ± 0.11, 0.68 ± 0.07, 0.41 ± 0.08, 0.54 ± 0.10, and 0.52 ± 0.07, respectively, as compared with controls. Exposure to a hot environment of 34 °C for 24 h led to an increase in transcript levels of *COI*, *COII*, *COIII*, *ND3*, *ND5*, *CYTB*, and *ATP6*, with values that were 3.3 ± 0.24, 2.0 ± 0.2, 2.70 ± 1.06, 1.57 ± 0,08, 1.47 ± 0.13, 1.39 ± 0.56, and 1.86 ± 0.12, respectively, over controls. By contrast, *ND4L* exhibited a significant decrease (to 0.31 ± 0.01) compared with controls. When exposed to 38 °C, the transcript levels of the 13 PCGs significantly increased, ranging from a 2.04 ± 0.23 increase in *ND1* to a 6.30 ± 0.96 rise in *ND6*. Under two different levels of cold and heat stress, the expression patterns of mitochondrial genes in *S. incognitus* vary, possibly associated with different strategies employed by this species in response to low and high temperatures, allowing for rapid compensatory adjustments in mitochondrial electron transport chain proteins in response to temperature changes. Furthermore, this underscores once again the significant role of mitochondrial function in determining thermal plasticity in reptiles.

## 1. Introduction

The ability of animals, especially ectotherms, to survive, develop, stay healthy, and reproduce depends crucially on the climate in which they live. Ecologists have long faced a formidable problem in anticipating how organisms will be affected by climate change and comprehending how they will respond to it [1]. Global temperatures are expected to continue rising, with an estimated 1.5 °C rise in the average global temperature between 2021 and 2040, according to the Sixth Assessment Report of the United Nations Intergovernmental Panel on Climate Change (IPCC) [2]. Modern climate change estimates predict a heightened frequency and intensified severity of extreme weather occurrences. Atypical occurrences, such as extended periods of extreme cold, heat waves, droughts, or exceptional rainfall, may have a significant influence on populations and could have long-term, significant effects on species and ecosystems [3,4,5]. Because of their limited capacity to utilize metabolic heat to control body temperature, ectotherms may be strongly impacted by climatic shifts [6]. Lizards, as a large group of reptiles, are typically ectothermic. Their body temperatures are mainly regulated by environmental temperatures, and their behaviors and physiological activities are directly constrained by temperature changes [7]. Frequent cold periods or heat waves affect their metabolic regulation and body temperature and can often necessitate a retreat into more thermally stable sites and/or alter the length of time available for activity and foraging. Lizards are highly likely to experience temperatures that exceed their minimum and maximum tolerances unless they can buffer environmental changes in some way [8].

Numerous biological activities, including energy metabolism, biosynthesis, signal transduction, Ca^2+^ homeostasis, immune response, and programmed cell death, depend on mitochondria [9,10]. But the activity of mitochondria is greatly impacted by temperature, and the capacity of organisms, particularly ectothermic animals, to maintain metabolic homeostasis and mitochondrial integrity in the face of temperature variations is essential [10,11,12]. Mitochondrial DNA (mtDNA) has higher copy numbers [13], a less efficient repair system [14], and higher vulnerability to damage [15], as compared to nuclear DNA (nDNA). Moreover, the cyclic GMP-AMP synthase (cGAS)-STING (stimulator of interferon genes) innate immune signaling pathway can be activated by the response to mtDNA stress [16,17]. Because of these special properties, mtDNA may serve as a sentinel for genotoxic stress in cells [18]. During stress responses, mtDNA can undergo changes earlier than nuclear DNA. The mitochondrial genome encompasses only 37 genes [19], which encode 13 essential proteins including 11 essential protein subunits of the mitochondrial electron transport chain and two subunits of the ATP synthase. These PCGs are fundamental components of the oxidative phosphorylation process, responsible for supplying 95% of an organism’s ATP energy [20,21]. The aerobic metabolic capacity of OXPHOS exhibits a positive correlation with the critical thermal limits (CTmax) across various ectothermic species, such as fish and invertebrates. This correlation underscores the pivotal role of mitochondrial functionality in determining thermal tolerance thresholds [12].

Scincidae (commonly called skinks) is the most species-rich family within the Sauria (http://www.reptile-database.org/, accessed on 29 January 2024) [22], but research on the mitochondrial response to climate change in this family is nearly non-existent. *Sphenomorphus incognitus* is known to inhabit forested pools as well as areas near rocks in slow-flowing streams and paths with abundant organic matter [23]. Currently, *S. incognitus* is distributed in northeastern Vietnam and southeast China. This species serves as a valuable model for researching how tiny and medium-sized lizards in subtropical and tropical areas react to challenges associated with cold or hot temperatures. In subtropical regions, when the temperature drops to 8 °C, these lizards enter hibernation. Research suggests that during hibernation, reducing energy expenditure and the consumption of internal fuel sources are mechanisms by which ectothermic animals lower their metabolic rate [24,25]. Metabolic and transcription activities in the body are strongly suppressed, and as the temperature drops to a certain extent, lizards may experience varying degrees of damage or even death. For example, after an extreme cold event at 4.4 °C, most lizards living in subtropical southern Florida were found frozen to death [26]. Additionally, in a study by He et al. [27], *Ateuchosaurus chinensis* (Chinese short-limbed skink) collected from Guangzhou, Guangdong, China, experienced mortality under laboratory conditions at 4 °C. Additionally, we noticed that the regulatory mechanism of mitochondrial respiratory chain complexes varies with temperature fluctuations, thereby promoting metabolic reshaping under changing environmental temperatures [28]. For instance, after 24 h in a low-temperature (freezing) state, the transcript levels of mitochondrial genes *16S RNA*, *ATP 6/8*, and *ND4* in North American wood frogs, *Rana sylvatica*, were significantly upregulated [29]. In a study by Wang et al., the expression of 10 mitochondrial PCGs in *Fejervarya kawamurai* was significantly downregulated after 24 h of exposure at 4 °C. However, when the temperature was lowered to 2 °C, the transcript levels of five genes increased [30]. Reactive oxygen species (ROS) are inevitable coproducts of energy metabolism, with the potential to cause significant damage to biological macromolecules. The excessive production of ROS resulting from oxidative stress is another adverse effect of low temperatures on the life history of ectotherms. To survive, organisms may reduce ROS production by increasing mitochondrial uncoupling or enhancing the activity of antioxidant enzymes to clear ROS [31]. In summary, we speculate that reptiles may adjust mitochondrial gene expression to cope with lower temperatures when exposed to low-temperature stress beyond the hibernation threshold.

The field of research concerning the prediction of potential future risks of thermal stress to worldwide biodiversity has become incredibly active. The rise in global temperatures presents a second challenge to organisms in the form of brief, intense heat waves [32]. In warmer environments, organisms exhibit higher energy metabolism. Increased mitochondrial activity also promotes faster development in ectotherms [33]. However, this does not necessarily imply lower levels of damage, since high temperatures can lead to the redistribution of electron flux and proton motive force (Δp), reducing ATP synthesis efficiency and increasing the relative cost of mitochondrial maintenance, resulting in excessive production of reactive oxygen species and raising the energy cost of antioxidant defense. This shift in mitochondrial energy economy may have negative impacts on organismal adaptive traits such as heat tolerance or growth [34]. In southern China, summer temperatures can reach between 32 °C and 40 °C (https://data.cma.cn/, accessed on 14 April 2024). Such high environmental temperatures increase the likelihood of heat stress in reptiles. If the energy expenditure in competing with thermal stress reaches a significant level capable of influencing reproductive output, the population may drop if exposed to abnormally high temperatures for a lengthy period of time or repeatedly [35]. Consequently, determining how organisms allocate mitochondrial energy resources becomes a crucial aspect of adaptation.

In this study, we simulated heatwaves and cold spells against a backdrop of global warming. We used 4 °C and 8 °C to simulate cold conditions, whereas 34 °C and 38 °C were used to simulate heat, with 25 °C set as the control temperature. Setting two low and two high temperatures allowed for a comparison of mitochondrial gene expression differences in lizards under varying degrees of cold and heat, thereby inferring lizard strategies in response to temperature fluctuations and their intrinsic connections with temperature changes. In addition, this study has provided new ideas for assessing the potential risks of global warming for skinks in China, as well as for understanding the response of mitochondrial genes in these species to climate change while maintaining energy balance.

## 2. Materials and Methods

### 2.1. Lizard Capture and Acclimatization

Fifty adult *S. incognitus* of similar size were captured from a creek and jungle in Xing’an County, northeast Guangxi Zhuang Autonomous Region, China (110°14′ to 110°56′ E and 25°17′ to 25°55′ N). All lizards were kept in 120 cm × 90 cm × 110 cm plastic incubators and acclimated to laboratory conditions at 25 °C for one week with a 12:12 h light/dark cycle. Daily sustenance consisted of live cockroaches or crickets, which were provided to the subjects on a twice-daily feeding schedule, ensuring they received fresh water daily for hydration. During this period, all lizards showed good activity levels and were able to feed voluntarily.

### 2.2. Experimental Design

In order to avoid the effects of lizard breeding, the experiment was conducted in the non-breeding season [36]. Five groups (10 lizards each) of acclimated lizards were created: extreme low-temperature exposure (4 °C), extreme moderate-low-temperature exposure (8 °C), extreme moderate-high-temperature exposure (34 °C), and extreme high-temperature exposure (38 °C). The control group was kept at a constant 25 °C. For 24 h, each group of 10 lizards (*n* = 50) was kept at the appropriate temperature in a temperature-controlled incubator. Treatment with low or high temperatures had no influence on the *S. incognitus* survival rate, as assessed at the conclusion of the experiment. Lizards were euthanized by decapitation [37], and 20 mg of liver tissue was harvested and transferred to RNA-free tubes. Harvested tissues were rapidly frozen in liquid nitrogen and stored at −80 °C for subsequent analysis of RNA extraction.

### 2.3. DNA Extraction, PCR, and Sequencing

We excised 2–3 mm of tail muscle from *S. incognitus* to extract DNA (without affecting the lizard’s normal life activities). Following the manufacturer’s protocol, an Ezup Column Animal Genomic DNA Purification Kit (Sangon Biotech Company, Shanghai, China) was used for DNA extraction. Universal primers [38] designed for lizards were used to amplify the 16S rRNA gene. The PCR products were sent to Youkang Company in Hangzhou, Zhejiang province, China, for sequencing using the bi-directional primer-walking method. We manually calibrated the returned 16S rRNA gene fragment sequence using SeqMan and then performed a BLAST comparison with data in GenBank. Results showed that these samples were over 99% consistent with the known sequence of *S*. *incognitus* (HQ132366) as reported in GenBank. The concentration of the mitochondrial genomic DNA of *S. incognitus* was measured using the Infinite M200pro enzyme label (Tecan, Ltd., Männedorf, Switzerland). Samples were sent to BGI Tech Inc. (Shenzhen, China) for next-generation sequencing once the concentration exceeded 25 ng/mL. The general process was as follows. Genomic DNA was randomly fragmented into uniform-sized pieces and adaptors were attached to the 5′ and 3′ ends. DNA fragments were then amplified via bridge PCR. Subsequently, paired-end sequencing of 150 bp was performed on the genomic DNA using the Illumina HiSeq 2000 platform. Finally, the size of the raw data ranged 7 GB, and its quality was assessed using FastQC. To ensure accurate assembly of the mitochondrial genome, two methods were utilized (NOVOPlasty v4.2 [39] and GetOrganelle v1.7.1 [40]), resulting in a successful assembly of the complete mitochondrial genome.

### 2.4. Mitochondrial Genome Localization and Sequence Analysis

We utilized the FASTA file of the 16,759 bp mitochondrial genome sequence attained from the lizards to identify the position of all tRNAs using the MITOS web server (http://mitos2.bioinf.uni-leipzig.de/index.py, accessed on 10 December 2023). Subsequently, we employed the reference genome of *S*. *incognitus* accessible in the NCBI to annotate the 13 PCGs, designating the initiation and termination codons, and verifying successful translation through MEGA7.0 [41]. Finally, we sequentially identified the positions of 12S rRNA and 16S rRNA. RSCU was directly obtained after importing the GB format file of the sequence into PhyloSuite v.1.2.3 [42] and extracting it. Mitochondrial genome maps were generated in the online website CG View (https://cgview.ca/, accessed on 15 December 2023) [43], where GC and AT skews were calculated based on the following formulas: AT skew = (A − T)/(A + T), GC skew = (G − C)/(G + C). The accession number is PP571909.

### 2.5. RNA Extraction and cDNA Synthesis

According to the manufacturer’s protocol, total RNA was extracted from liver tissues of *S. incognitus* from different temperature groups using an RNA extraction kit from Chengdu Fuji Biological Company (Chengdu, China). Total RNA was scrutinized for quality via 1% agarose gel electrophoresis. The concentration of total RNA was determined with an infinite M200pro enzyme label (Tecan, Ltd., Männedorf, Switzerland), whereas the A260/280 ratio (1.8~2.1) was used to appraise RNA purity. During the process of RNA to cDNA conversion, the system for the first step was mixed to a final volume of 10 μL using the PrimeScript^™^ RT reagent Kit with gDNA Eraser (Takara, Japan) according to the manufacturer’s instructions: 2 μL 5X gDNA Eraser Buffer and 1 μL gDNA Eraser were mixed with 7 μL of a mixture of RNase-Free ddH_2_O and RNA. The purpose of this mixed system was to remove genomic DNA by heating at 42 °C for 2 min. The reaction system for the second step comprised a 10 µL mixture, composed of 1 µL PrimeScript RT Enzyme Mix I, 1 µL RT Primer Mix, 4 µL 5X PrimeScript Buffer 2, and 4 µL RNase-free ddH_2_O. After mixing the systems from the two steps, a thermal cycling program was operated according to the following protocol: 37 °C for 15 min and 85 °C for 5 s. The synthesized cDNA was then stored at −80 °C.

### 2.6. RT-qPCR Primer Design and Screening

Quantitative primers were designed for the 13 PCGs of *S. incognitus* using Primer Premier 6.0 software (Premier Biosoft International, Palo Alto, CA, USA). The design criteria for the primers included an amplified fragment length of approximately 100–150 base pairs, a primer length of 18–22 nucleotides, a primer melting temperature (Tm) of 50–55 °C, and a GC content of approximately 50%.

EASY Dilution was added to the cDNA, diluting samples into five different concentrations for use in *Ct* value (threshold cycle) measurement in real-time fluorescent quantitative PCR. The primers were designed for use in real-time PCR and their suitability was tested using the StepOnePlus™ PCR system (Life Technologies, Carlsbad, CA, USA). Available quantitative primers obtained after screening are listed in Table 1. The upstream primer for amplifying *β-actin* was GATCTGGCATCACACTTTCT, and the downstream primer was GTGACACCATCACCAGA [44,45]. The *β-actin* gene served as an internal reference for standardization of target genes [46].

### 2.7. Relative mRNA Quantification

The StepOnePlus™ Real-Time PCR System (Life Technologies, Carlsbad, CA, USA) was utilized to perform reverse transcription–quantitative polymerase chain reaction (RT-qPCR) and determine the relative mRNA expression levels of the 13 PCGs in the liver mitochondria of *S. incognitus* exposed to various temperatures. Each PCR reaction was performed in a total volume of 20 μL, including 6 μL ddH_2_O, 10 μL SYBR Premix Ex Taq II (2×), 2 μL cDNA, 0.4 μL ROX Reference Dye (50×), and 0.8 μL of each forward and reverse primer. Each sample’s qPCR was conducted in triplicate according to the following conditions: an initial denaturation at 95 °C for 30 s, followed by 40 cycles of denaturation at 95 °C for 5 s, and annealing at 55 °C for 30 s. Melting curve analysis was performed at 95 °C,15 s, 60 °C, 1 min, 95 °C, 15 s.

### 2.8. Data Analysis

The Statistical Program for Social Sciences 22.0 software (SPSS, Inc., Chicago, IL, USA) was used for statistical analysis, and the 2^−ΔΔCt^ method was used for calculations. All data were expressed as mean ± SE. To reduce errors caused by RNA quantity, quality, or reverse transcription efficiency, the expression values of the reference gene *β-actin* (not influenced by experimental treatments) were used to standardize the expression levels of each target gene in each sample. Grubbs’ test for outliers was performed with a 95% confidence level. Student’s *t*-test was used to compare gene transcript levels in the liver between control and experimental groups, and a difference was considered statistically significant when *p* < 0.05. Graphs were generated using Origin 2021 software (Origin Lab, Northampton, MA, USA) to visually illustrate the data.

## 3. Results

### 3.1. General Features of the Sphenomorphus incognitus Mitogenome

**Figure 1:**
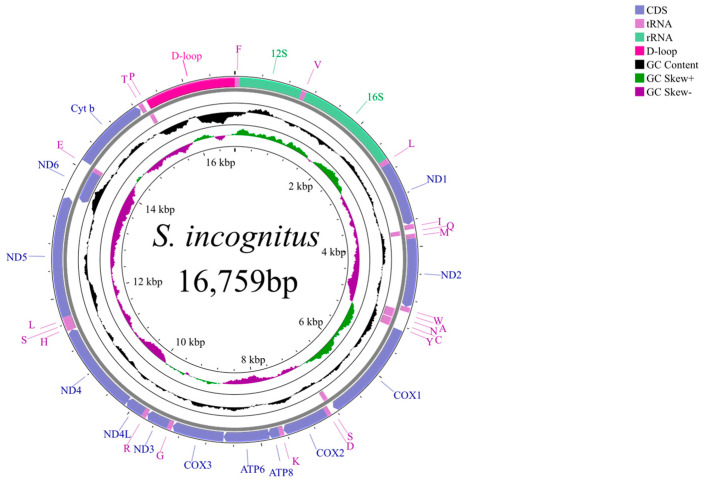
The complete mitochondrial genome map of *S. incognitus*. The outer circle shows the arrangement of genes. External genes are encoded by the positive strand (5′ → 3′), and internal genes are encoded by the negative strand (3′ → 5′). The second circle shows GC content. The innermost circle shows GC skew.

*S. incognitus* possesses a complete mitochondrial genome with a length of 16,579 bp. This genome is composed of 13 PCGs spanning 11,385 bp, 22 tRNA genes covering 1159 bp, 2 rRNA genes (12S rRNA and 16S rRNA) measuring 938 bp and 1540 bp, respectively, and a control region spanning 1137 bp. A comprehensive overview of the specific genomic locations can be seen in Table 2. Notably, the *ND6* gene and eight tRNA genes are located on the positive chain, whereas the remaining genes reside on the negative chain (Figure 1).

**Figure 2:**
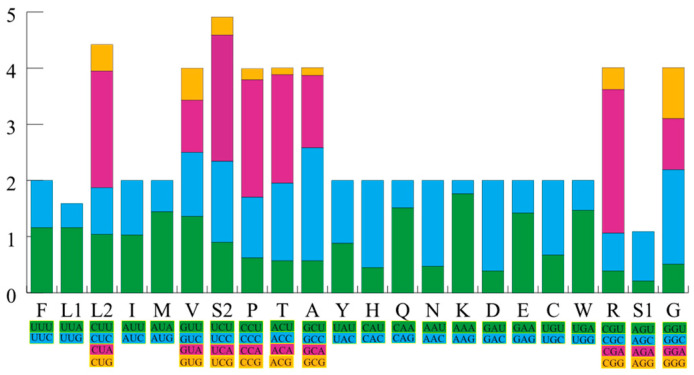
The relative synonymous codon usage (RSCU) of the mitochondrial genome of *S. incognitus.* The *X*-axis depicts the codons employed along with their various combinations of synonymous codons, represented by different colors. The *Y*-axis presents the RSCU values.

The detailed nucleotide composition of the 37 genes, providing the respective percentages of A, T, C, and G, along with the calculated AT and GC skews, is presented in Table 3. Notably, upon conducting a thorough composition analysis, it was discovered that the mitochondrial genome of *S. incognitus* consists of A (30.9%), T (25.8%), C (28.2%), and G (15.0%). Furthermore, it is worth mentioning that the combined content of A and T is markedly greater than that of C and G. From the RSCU of the 13 PCGs of *S. incognitus*, it can be observed that the most frequently used codon is CUA (Leu) with a count of 228, whereas the least used codons are CGU (Arg) and CGG (Arg), with counts of 7 each (Figure 2). The mitochondrial genome of *S. incognitus* encodes 3785 amino acids, excluding stop codons.

Overall, the mitochondrial genome of *S. incognitus* is generally similar to that of other skinks in terms of structure and traits, with no particular exceptions.

### 3.2. Quantification of Mitochondrial Protein-Coding Genes at Low Temperatures

**Figure 3:**
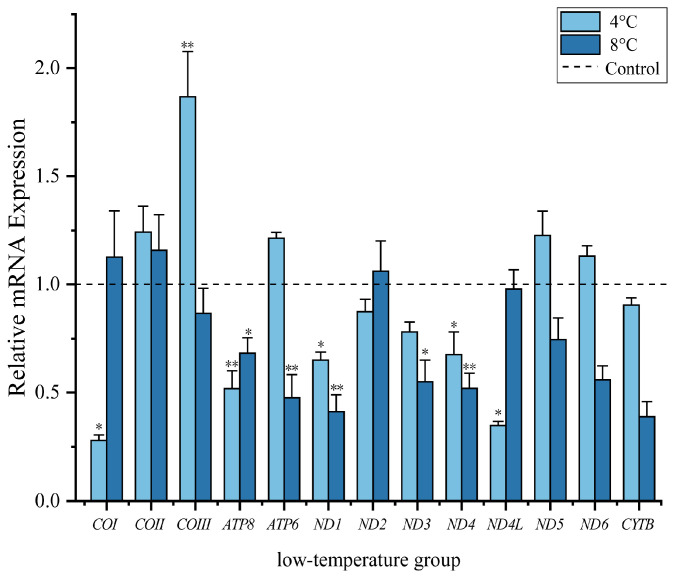
Transcript levels of 13 PCGs in lizards were assessed under low-temperature stress, as compared to 25 °C values. The bars represent the mean ± standard deviation of the original data. “*” indicates a significant difference between different treatment groups and controls, *p* < 0.05 (*), *p* < 0.01 (**).

The relative transcript level changes of the mRNA for the 13 PCGs in *S. incognitus* under acute low-temperature stress, compared to the control group (25 °C), are illustrated in Figure 3. Under the extreme moderate-low-temperature (8 °C) condition, the expression levels of nine PCGs exhibited a decreasing trend. Specifically, transcript levels of *ATP8* and *ND3* were significantly reduced (*p* < 0.05) to levels of 0.68 ± 0.07 and 0.54 ± 0.10, respectively, as compared to control values. *ATP6*, *ND1*, and *ND4* showed significant levels of reduction (*p* < 0.01), with levels dropping to just 0.48 ± 0.11, 0.41 ± 0.08, and 0.52 ± 0.07, respectively, as compared with controls. Under the extremely low temperature (4 °C) condition, significant decreases in the expression levels of several genes were noted. As compared with controls, *ATP8* transcript levels fell to 0.52 ± 0.08, *ND1* transcripts decreased to 0.65 ± 0.04, and *ND4* was reduced to 0.68 ± 0.10 (*p* < 0.05). By contrast, there was a significant increase in the transcript level of *COIII*, with a fold change of 1.86 ± 0.21 (*p* < 0.01). At the same time, transcript levels of *COI* and *ND4L* genes also showed statistically significant reductions compared to the control group (*p* < 0.05), with levels falling to 0.28 ± 0.02 and 0.35 ± 0.02, respectively, as compared with controls.

### 3.3. Quantification of Mitochondrial Protein-Coding Genes at High Temperatures

**Figure 4:**
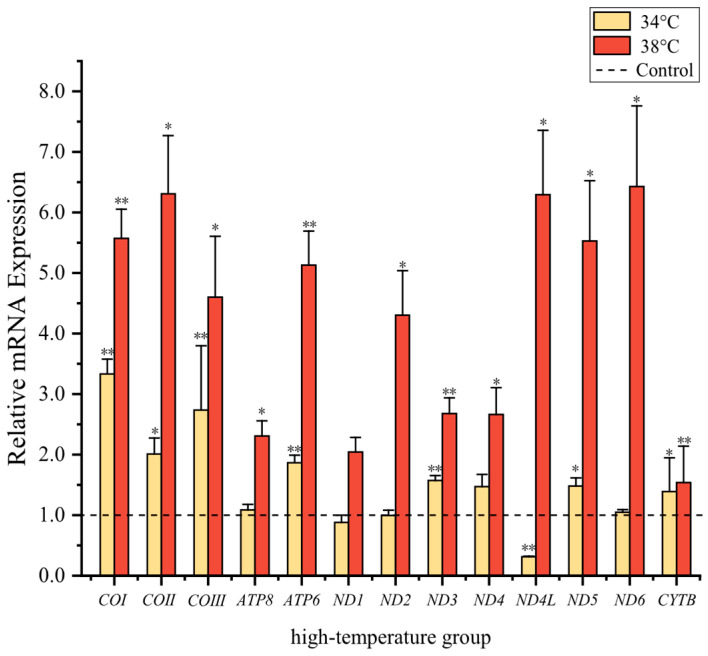
Transcript levels of 13 PCGs in lizards were assessed under high-temperature stress, as compared to 25 °C values. The bars represent the mean ± standard deviation of the original data. “*” indicates a significant difference between different treatment groups and controls, *p* < 0.05 (*), *p* < 0.01 (**).

The high-temperature and low-temperature groups displayed distinct trends. At 34 °C, an extreme moderate-high temperature, *COII*, *ND5*, and *CYTB* transcript levels increased significantly by 2.0 ± 0.27, 1.47 ± 0.133, and 1.39 ± 0.56, respectively, as compared with the control group at 25 °C (*p* < 0.05). In mitochondrial complex IV, *COI* and *COIII*, complex I (*ND3*) as well as *ATP6* in complex V, showed strong significant increases with values of 3.3 ± 0.24, 2.7 ± 1.06, 1.57 ± 0.08, and 1.86 ± 0.12, respectively, as compared to the control group (*p* < 0.01). By contrast, the transcript level of *ND4L* in mitochondrial complex I showed a strong significant decrease to a value of just 0.31 ± 0.01 (*p* < 0.01), as compared to controls. When the temperature rose further to 38 °C, there was no longer a decline in gene expression. All 13 PCGs in mitochondria displayed upregulation with strong significant changes (Figure 4). Mitochondrial gene transcripts of *ND1*–*ND6*, *COI*–*COIII*, *ATP8*, *ATP6*, and *CYTB* increased to 2.04 ± 0.23, 4.30 ± 0.73, 2.68 ± 0.26, 2.67 ± 0.44, 6.29 ± 1.06, 5.53 ± 1.00, 6.43 ± 1.33, 5.57 ± 0.48, 6.30 ± 0.96, 4.60 ± 1.00, 2.30 ± 0.25, 5.13 ± 0.57, and 1.54 ± 0.60, respectively.

## 4. Discussion

### 4.1. Analysis of Mitochondrial Transcript Levels at Low Temperature

As predicted, the transcript patterns of mitochondrial gene expression in *S. incognitus* were different under the two levels of cold stress as compared to the control group. In this study, after experiencing cold stress at 8 °C, the transcript levels of *ATP8*, *ND3*, *ATP6*, *ND1*, and *ND4* showed significant decreases. The subunits encoded by the *ATP6* and *ATP8* genes are subunits of the F_o_, F_1_-ATPase, which is responsible for proton flow through the Fo, F1-ATPase, leading to ATP generation via oxidative phosphorylation. *ND1*, *ND3*, and *ND4* are crucial components of the hydrophobic subunits within the membrane arm of Complex I. The subunits play a pivotal role in the initial stages of complex assembly, as well as exerting control over the regulation of complex I, which encompasses the control of protein degradation rates [47]. The decrease in transcript levels of mitochondrial genes reduces the consumption of endogenous fuel supply required for ATP production and may be a physiological state where ectothermic animals actively suppress metabolism, lower body temperature, and slow down other life processes to conserve energy and survive under cold environmental conditions [48]. Even at lower temperatures, reaching 4 °C, Complex I (ND1, ND4) remained inhibited.

In previous studies examining mitochondrial gene expression and positive selection analysis in relation to low temperatures, particularly in insects [49], amphibians [50], and reptiles [51], the majority of identified genes were found to be components of mitochondrial Complex I. Within mitochondria, the proteins that these genes encode play crucial roles in the tricarboxylic acid cycle (TCA cycle) and the NADH generated from beta-oxidation. They are also involved in the decreasing of ubiquinone and the transhipment of protons across through the inner membrane, contributing significantly to the proton motive force. Remarkably, the ND complex alone is responsible for a considerable proportion of total energy generation within the mitochondria. Moreover, research has demonstrated that the activity of Complex I is constrained under low temperatures [34,52]. In order to counteract the negative effects of low temperatures on enzyme reaction rates, these organisms can make adjustments to their biochemical processes, allowing them to adapt to cold temperatures. In certain instances, this adaptation can lead to full functional compensation in intact animals [53]. Interestingly, mitochondrial Complex I was found to have regulatory functions at extremely low temperatures of 4 °C and 8 °C, suggesting that *S. incognitus* has evolved an energy metabolism adaptation to extreme environments by modifying the efficiency of NADH dehydrogenase. In a study of cold adaptation in *Calotes versicolor* (the oriental garden lizard), similar regulatory functions of Complex I were found [27].

At 4 °C, besides the decrease in transcript levels of Complex I (*ND1*, *ND4*) and Complex V (*ATP8*), Complex IV joined the regulatory process, but transcript levels of *COIII* increased. Similarly, we observed a rapid increase in transcript levels of *COIII* in *A*. *chinensis* after cold stress [27]. Low temperatures can disturb membrane structure and abundance of mitochondria, leading to the production of ROS [54]. Excessive production of ROS can induce oxidative modifications to cellular macromolecules, thereby inhibiting protein function, upregulating the expression of pro-apoptotic proteins, and triggering cell apoptosis [55]. To mitigate the impact of ROS, organisms often increase the production of antioxidants, such as superoxide dismutase, to prevent oxidative damage to protein synthesis or protein hydrolysis [56]. Glycolysis has been shown to be downregulated in species of the genus *Takydromus* in response to cold acclimation [57]. Alternatively, as observed in other ectothermic species, they may increase glucose and urea concentrations as cryoprotectants [58]. The activation of antioxidants, glycogen breakdown in liver cells, and the generation of cryoprotectants or antifreeze agents require ATP. However, the inherent production capacity under low metabolic conditions can be insufficient to meet the additional energy demands. This suggests that the increase in mitochondrial *COIII* transcript levels at 4 °C in *S. incognitus* served as a compensatory metabolic mechanism to maintain ATP supply and demand under low metabolic conditions. Therefore, in the face of ROS-induced oxidative damage and during other metabolic activities (e.g., glycolysis) being inhibited, the maintenance of basic metabolism may be a contributing factor to the elevated expression levels of mitochondrial PCGs at extremely low temperatures.

Although ectothermic animals are highly susceptible to the effects of climate change, many possess the capacity to seasonally enter a state of metabolic depression or employ metabolic compensation strategies to survive in cold environments [59,60,61]. Indeed, a study of *F. kawamurai* by Wang et al. [30] showed a decrease in transcript levels of 10 mitochondrial PCGs at 4 °C. When temperature dropped to 2 °C, *ND5* maintained reduced levels whereas the expression of six PCGs significantly rose compared to controls. Similar to our results, this suggests that undergoing varying degrees of low temperature first induces dormancy and then triggers a stress response. The response strategies of mitochondrial PCGs in amphibians and reptiles vary under different degrees of extreme cold. This also suggests commonalities in the application of mitochondrial genes for cold resistance in amphibians and reptiles. Based on our study, at a low temperature of 8 °C, *S. incognitus* employed a primarily metabolic depression strategy by downregulating the transcript levels of mitochondrial PCGs as an adaptation to the cold. Conversely, at a low temperature of 4 °C, these lizards utilized both metabolic depression and metabolic compensation strategies to adapt to the cold.

### 4.2. Analysis of Mitochondrial Transcript Levels at High Temperature

Warmer environments promote faster development and increased metabolic activities in poikilothermic animals, implying higher energy demands [33]. Consistent with this pattern, transcript levels of seven PCGs showed upregulation under 34 °C conditions. Similarly, transcript levels of the 13 PCGs significantly increased in *S. incognitus* at 38 °C. Functions that ATP synthase and the electron transport chain Complex I, III, and IV played in maintaining normal activity and meeting energy needs in high-temperature environments were essential to mitochondrial operation.

At 34 °C, the expression of the *ND4L* gene was downregulated, and a possible explanation for this phenomenon was that it was linked to a reduction in transcript levels of the gene in *S. incognitus*, potentially leading to a decrease in ROS release. Salin [62,63] and colleagues also observed low levels of ROS under a high standard metabolic rate (SMR), which was achieved by increasing mitochondrial uncoupling. It is known that complex I is the main generator of ROS in environments that encourage reverse electron transport [64]. The protein encoded by the *ND4L* gene is a key subunit of complex I in the electron transport chain and is involved in its fundamental function [65]. A downregulation of *ND4L* gene expression also occurred in mayflies as a means to reduce ROS production [66]. Consequently, the marked decline in the *ND4L* gene steady-state transcript levels may act as a useful strategy for cells to lessen ROS production at its source.

There was a downregulation of genes at 34 °C, but this did not occur at the higher temperature of 38 °C. We proposed that, in contrast to the adaptation to gradual long-term temperature changes, organisms exposed to extremely high temperatures had a transient increase in individual heat tolerance. This increase was primarily attributed to the production of heat shock proteins (HSP70) [67]. The temperature change for *S. incognitus* from 34 °C to 38 °C necessitated a higher heat tolerance, resulting in the allocation of more energy towards costly physiological activities, such as the activation of heat shock proteins. This shift in energy allocation could lead the lizards to not downregulate gene expression in order to reduce ROS production. Instead, they generated more energy to increase antioxidant capacity for ROS clearance and activated heat shock proteins to expand the maximum heat tolerance limit.

It is crucial to note that increased energy metabolism in warm environments does not necessarily indicate low levels of damage. On the one hand, elevated temperatures amplify the occurrence of events linked to increased oxidative stress [31,68]. On the other hand, heightened energy metabolism comes at a cost [69]. Higher temperatures can impede the functioning of the mitochondrial respiratory chain, resulting in excessive generation of ROS and subsequent oxidative damage [70]. In addition, prolonged exposure to high temperatures can escalate proton leakage, including uncoupling protein-mediated proton leakage, which often increases at a steeper rate than the rate of oxidative phosphorylation [34]. One notable repercussion of this leakage is its potential to diminish the ATP conversion rate. Inefficient proton cycling and heightened ROS formation gradually erode energy efficiency, thereby impacting organismal function and lifespan. Some researchers hold the belief that the antioxidant defense mechanism, involving the enhancement of antioxidative enzymes like SOD, GSH-Px, and CAT, might exhibit a positive correlation with energy metabolism [71]. In this investigation, as the ambient temperature rose, so did the upregulation of transcript levels of mitochondrial PCGs. Whereas direct measurement of ROS production was not conducted in this study, the augmented number of upregulated genes serves as an indication that acute heat stress disrupted the equilibrium between the antioxidant system and ROS production. Heat-induced ROS elevation can damage organelle membranes [72,73]. The oxygen- and capacity-limited thermal tolerance (OCLTT) hypothesis also suggests that efficiency can be influenced with the inability to supply sufficient oxygen to respiratory mitochondria, leading to thermal limitations caused by an imbalance between oxygen availability and demand [74,75,76,77]. Consequently, the transcript levels of mitochondrial genes were shown to have increased significantly in order to help counteract the action of the mitochondrial respiratory chain complexes, provide more energy to antioxidant mechanisms, and regulate mitochondrial inner membrane proton permeability. This implies that mitochondrial activity has a significant impact in determining heat tolerance limitations.

## 5. Conclusions

In the present study, we noticed distinct cold adaptation strategies of *S. incognitus* under two different temperatures. At 8 °C, transcript levels of *ATP8*, *ND1*, *ND3*, *ND4*, and *ATP6* genes were significantly decreased, indicating a strategy of metabolic inhibition. Under 4 °C stress, transcript levels of *ATP8*, *ND1*, *ND4*, *ND4L*, and *COI* genes were also significantly reduced, whereas the transcript level of *COIII* showed a significant increase, suggesting a combined strategy of metabolic depression and compensation. Moreover, under high-temperature stress, *S. incognitus* showed increased transcript levels of *COI*, *COII*, *COIII*, *ND3*, *ND5*, *CYTB*, and *ATP6* at 34 °C, while reducing the transcript level of *ND4L*. At 38 °C, increased transcript levels of all 13 mitochondrial PCGs were found. Organisms can adjust their minimum and maximum thermal tolerance by regulating transcript levels of mitochondrial genes at different temperatures. Further investigation of the physiological changes in *S. incognitus* under low- and high-temperature stresses, as well as changes in various heat acclimation indicators, will provide a clearer understanding of the findings presented in this study.

## Figures and Tables

**Table 1:** *RT-qPCR* primers for the 13 mitochondrial PCGs and *β-actin* in this study.

Gene Name	Forward Primers (5′-3′)	Reverse Primers (5′-3′)
*COI*	GXXA-COI-J1	GXXA-COI-N1
	GCATTGTCCTAGCCAATTCATC	AACATTACGCCGAAGTGGATT
*COII*	GXXA-COII-J1	GXXA-COII-N1
	ATCGCCCTTCCCTCACTTC	TCGTAGTCTGTGTATTCGTAGC
*COIII*	GXXA-COIII-J1	GXXA-COIII-N1
	CCACCAAGCACACGCATAC	GGTAATGTCTCGTCATCACTGT
*ATP8*	GXXA-ATP8-J1	GXXA-ATP8-N1
	CTGAGCCACAATTCTTCTAATC	TAGGTTCATGGTCAGGTTCA
*ATP6*	GXXA-ATP6-J1	GXXA-ATP6-N1
	TTGTGGCTGTCTACTGTCCTTA	CGGTTAGGTTGGCGGTTAGT
*ND1*	GXXA-ND1-J1	GXXA-ND1-N1
	GACTACTATTCTGCTCGTGACC	AATGTTGGCGTATTCGGCTAG
*ND2*	GXXA-ND2-J1	GXXA-ND2-N1
	ACCTGAGACATTACACAACTGA	TGTAGGACCTCTGGCAATCA
*ND3*	GXXA-ND3-J1	GXXA-ND3-N1
	GCTTCCGTTCTCACTTCGTT	GGCTGCTCACTCGTATACTAAG
*ND4*	GXXA-ND4-J1	GXXA-ND4-N1
	AACCTTCACCATCAACTCTTCC	TAGGAGCCAGCAGGATAGAAC
*ND4L*	GXXA-ND4L-J2	GXXA-ND4L-N2
	GGCATCCTCGGCTTATCAATAC	GGAGCATAGTTGTGGAGGGTAA
*ND5*	GXXA-ND5-J1	GXXA-ND5-N1
	CAGCATACAGTCTACGGTTGAT	AAGGCTAGGCGGAGGATTG
*ND6*	GXXA-ND6-J1	GXXA-ND6-N1
	CCCAAGAGTAAAGCAAAGAGAT	GTGTTGTTGTTGTTCGTGTT
*Cytb*	GXXA-CYTB-J1	GXXA-CYTB-N1
	TCTGCCGAGATGTTCAATATGG	GACGAAGGCTGTTGCTATTACT
*β-actin*	GXXA-ACT-J1	GXXA-ACT-N1
	GCCATGTACGTTGCCATCC	CCAGAGTCCATCACGATACCA

**Table 2:** Locations of features in the mtDNA of *S. incognitus*.

Gene/Region	Start Position	Stop Position	Spacer (+) Overlap (−)	Length (bp)	Start Codon	Stop Codon	Strand
tRNA^Phe^	1	72		72			H
12S rRNA	73	1010		938			H
tRNA^Val^	1011	1081		71			H
16S rRNA	1082	2621		1540			H
tRNA^Leu (UUR)^	2622	2697		76			H
ND1	2698	3660	+1	963	ATG	TAA	H
tRNA^Ile^	3662	3734		73			H
tRNA^Gln^	3735	3806	−2	72			L
tRNA^Met^	3805	3874		70			H
ND2	3875	4909		1035	ATG	TAA	H
tRNA^Trp^	4910	4981	−1	72			H
tRNA^Ala^	4981	5049		69			L
tRNA^Asn^	5050	5122	+13	73			L
tRNA^Cys^	5136	5199		64			L
tRNA^Tyr^	5200	5266	+1	67			L
COI	5268	6815	−5	1548	GTG	AGA	H
tRNA^Ser (UCN)^	6811	6883	+4	73			L
tRNA^Asp^	6888	6955		68			H
COII	6956	7643		688	ATG	T	H
tRNA^Lys^	7644	7708	+2	65			H
ATP8	7711	7878	−10	168	ATG	TAA	H
ATP6	7869	8552	−1	684	ATG	TAA	H
COIII	8552	9335		784	ATG	T	H
tRNA^Gly^	9336	9405		70			H
ND3	9406	9751		346	ATG	TAA	H
tRNA^Arg^	9752	9820		69			H
ND4L	9821	10,117	−7	297	ATG	TAA	H
ND4	10,111	11,491		1381	ATG	T	H
tRNA^His^	11,492	11,563		72			H
tRNA^Ser (AGY)^	11,564	11,629	−1	66			H
tRNA^Leu (CUN)^	11,629	11,701	+1	73			H
ND5	11,703	13,529	−5	1827	ATG	TAA	H
ND6	13,525	14,046		522	ATG	AGG	L
tRNA^Glu^	14,047	14,115	+2	69			L
Cyt *b*	14,118	15,260	+6	1143	ATG	TAA	H
tRNA^Thr^	15,267	15,335		69			H
tRNA^Pro^	15,336	15,404		69			L
D-loop	15,405	16,759		Complete			H

**Table 3:** Composition of the mitochondrial genome of *S. incognitus*.

Region	Strand	Length (bp)	A (%)	T (%)	C (%)	G (%)	A + T (%)	C + G (%)	AT Skew	GC Skew
Whole genome		16,759	30.9	25.8	28.2	15.0	56.7	43.2	0.090	−0.307
PCGs	+	10,860	28.9	27.5	29.4	14.2	56.4	43.6	0.025	−0.347
	−	522	9.6	40.8	11.7	37.9	50.4	49.6	−0.620	0.529
tRNAs	+	986	31.5	25.4	23.3	19.8	56.9	43.1	0.109	−0.082
	−	556	25.9	29.7	16.0	28.4	55.6	44.4	−0.068	0.279
rRNAs	+	2478	35.6	20.9	25.3	18.3	56.5	43.6	0.260	−0.161

## Data Availability

Data to support this study are available from the National Center for Biotechnology Information (https://www.ncbi.nlm.nih.gov, accessed on 2 April 2024). The GenBank number is PP571909.
